# Ingested a fish bone-induced ileal perforation: A case report

**DOI:** 10.1097/MD.0000000000019508

**Published:** 2020-04-10

**Authors:** Junchuan Song, Weijin Yang, Yuewen Zhu, Yongchao Fang, Jiandong Qiu, Jianshen Qiu, Lan Lin, Weihang Wu, Chen Lin, Yu Wang

**Affiliations:** a Department of General Surgery, Dongfang Hospital, Xiamen University; b Department of General Surgery, 900 Hospital of the Joint Logistics Team; c Clinical Institute of Fuzhou General Hospital, Fujian Medical University; d Interventional ward of medical imaging center, 900 Hospital of the Joint Logistics Team, China.

**Keywords:** fish bone, foreign body, ileum

## Abstract

**Introduction:**

Gastrointestinal perforation due to foreign body intake is rare and often secondary to unintentional intake; hence, a misdiagnosis is likely. Herein, we report a case of perforation of the ileum due to fish bone.

**Case presentation:**

A 57-year-old woman presented with right lower abdominal pain. She did not provide any information about having a history of swallowing foreign bodies. Surgery for uterine fibroids and subtotal gastrectomy was performed 6 years ago.

**Diagnosis:**

Laboratory tests and imaging examination showed normal results. During laparotomy, a fish bone was found at the end of the ileum. Two senior radiologists re-evaluated the computed tomography scan, and confirmed the presence of the suspected foreign body.

**Interventions:**

Partial intestinal resection and manual ileum end anastomosis were performed.

**Outcomes:**

The patient recovered well after surgery and recalled that she had eaten fish the night before experiencing abdominal pain.

**Conclusion:**

An accurate diagnosis of complications due to fish bone intake, often secondary to the unintentional intake, is quite challenging. Detailed history-taking about the patient's diet and eating habits is therefore important. Clinical manifestations are mainly determined by the location of perforation, which typically occurs at the junction of the ileum and rectal sigmoid colon. Imaging examination and surgery are often used for definite diagnosis.

## 1 Introduction

Foreign body intake such as dentures, toothpicks, fish bones^[1]^ is a common phenomenon, but perforation caused by foreign bodies is rare, and only 1% of gastrointestinal perforation is due to foreign body intake. The fish bone is the most common object that causes perforation of the gastrointestinal tract.^2^,^3^ Given that only few patients can recall foreign body intake, differences in clinical performance and the low sensitivity of imaging examination increases the difficulty of arriving at a correct diagnosis. Most patients need to surgery to diagnose and detect intestinal foreign bodies.^[4]^ Literature states that early surgical interventions help to diagnose and remove parts of the intestine, given the possibility of abscess formation and delayed complications due to fish bone movement.^[5]^ Herein, we report a case of perforation at the end of the ileum caused by fish bone ingestion.

## 2 Case report

**Figure 1:**
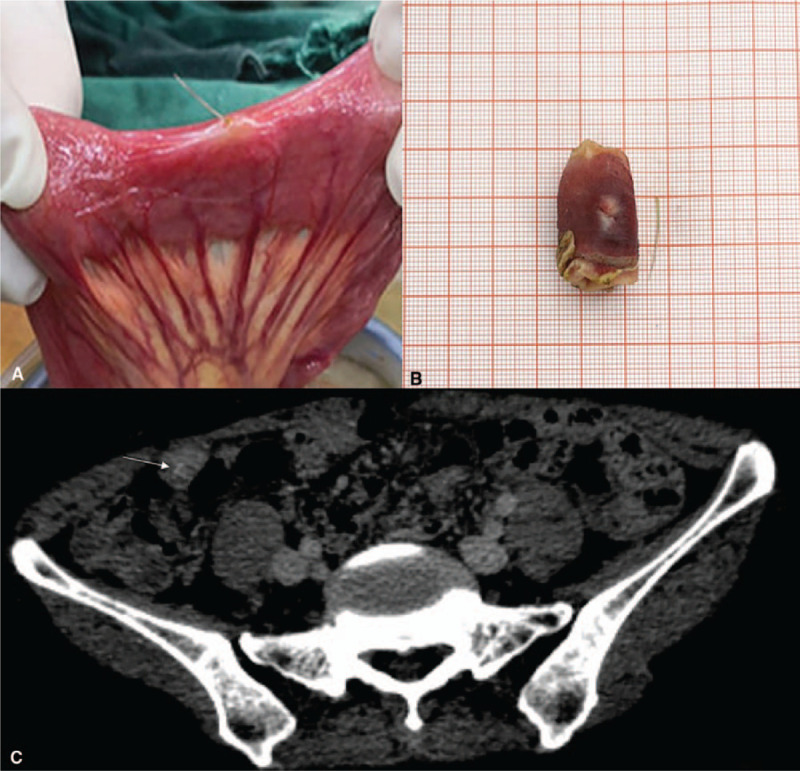
A. Operative findings showed the fish bone lodged at the end of the ileum causing perforation and coated with a small amount of fibrinous exudates. There was no definite obstruction or stricture. B. The distal ileum was removed along with the 1.7-cm-long fish bone. C. Re-evaluation with contrast-enhanced abdominal CT showed a suspected radiopaque linear shadow at the end of the ileum lodged into the thickened intestinal wall. There was no evidence of localized inflammation or fluid collection. (white arrow).

A 57-year-old woman was admitted to the general surgery department with complaints of right lower abdominal pain that started 3 days ago. The patient showed no symptoms of peritonitis, fever, and other inflammatory aspects. Her white blood cell count, blood urea nitrogen level, and defecation were normal. Surgery for uterine fibroids and subtotal gastrectomy was performed 6 years ago. Contrast-enhanced abdominal computed tomography (CT) did not show any obvious abnormalities. As the patient had a history of multiple surgeries, we wondered whether the pain was due to postoperative intestinal adhesion. However, on the third day after hospital admission, we decided to perform a laparoscopic exploration. There was a small amount of yellow exudate in the pelvic cavity. Given the patient's history of multiple abdominal surgeries, part of the intestinal showed adhesion, and the laparoscopy could not provide a comprehensive exploration. We surgically opened the abdomen and found a sharp, hard foreign body measuring 1.7 cm at the end of the ileum. It was coated in some fibrinous exudates around the puncture point (Fig. 1A). Finally, partial intestinal resection and manual ileum end anastomosis were performed (Fig. 1B). Two senior radiologists re-evaluated the CT scan, and an image of the suspected foreign body was found. There was no evidence of localized inflammation or fluid collection (Fig. 1C). The patient's medical history was re-enquired after surgery when she recalled that she had eaten a fish called *Argyrosomus argentatus.* Pathological examination of surgical specimens showed acute inflammation, and the patient was discharged normally on the 7th day.

## 3 Discussion

Although most foreign bodies can be extruded within one week after entering the digestive tract,^[1]^ 1% of patients may still have perforations due to long or sharp fish bones. It is difficult for doctors to make a clear diagnosis, because patients cannot recall the history of foreign body intake and are thus often misdiagnosed as appendicitis or perforation of digestive tract ulcers.^[6]^ Depending on the location of the damage, clinical manifestations often vary, including constipation, abdominal pain, anal pain, abscesses, and anal fistula. It is reported that 95%patients present with abdominal pain as the most important symptom, 81% patients develop fever, and 39% have local peritonitis.^[7]^

Most foreign bodies leading to perforation of the gastrointestinal tract are caused by eating foods such as sharp broken bones and fish bones. In one study, fish bones were found to be the most common foreign bodies that lead to perforation of the gastrointestinal tract. In certain countries or regions that prefer to eat fish, gastric perforation or other complications caused by fish bone intake are very common.^[8]^

Although perforation caused by fish bones can affect any part, it is most often seen occurring in areas of physiological stenosis or intestinal transitions such as the ileum or rectosigmoid junction.^[6]^ In a previous report, the probability of ileal perforation is 83%.^[9]^ In another article, the perforation of the end of the ileum accounted for 38.6%, while that of the jejunum was lower, accounting for only 14.3%.^[1]^

Ingestion of fish bones leading to perforation is often secondary to accidental intake. Therefore, doctors rarely obtain a history of fish-bone ingestion. It is often only found during an imaging examination or surgical exploration.^[8]^

Imaging examination is usually unreliable in the diagnosis of perforation caused by fish bone. High-density shadow, free gas, and abscess formation are often used to determine the presence of inflammatory changes or perforation.^[10]^ At the same time, the fish bone gradually penetrates the intestinal wall through extrusion. In this process, the perforation site is often covered by fibrin or adjacent intestines that limits the outflow of intestinal contents and also reduces the chance of free gas appearance.^[2]^ Radiographic evidence of free intestinal gas was found in only 20% patients.^[9]^ In another study that analyzed 358 patients with fish bone perforation, X-rays were found to be only 32% sensitive.^[11]^ The fish bone is often disturbed by radiation doses and adjacent inflammatory tissues or liquids.^[12]^ CT determination of perforated areas is often achieved by identifying a thickened bowel segment, local effusion, fat infiltration, or any combination of these findings.^[13]^ However, these findings are less specific as compared to the diagnosis made by identifying high-density images caused by the impacted fish bone. In our case, we only used CT examination because X-rays are typically inadequate to reveal intestinal adhesion or complications after multiple surgeries. The preoperative CT examination did not reveal adequate information for a clear diagnosis, such as high-density shadow, local bowel thickening, or presence of air outside the lumen. After the operation, 2 senior radiologists re-evaluated the CT scan, when an image of the suspected foreign body was found; however, there was no evidence of localized inflammation or fluid collection.

This patient had previously undergone resection of uterine fibroids and subtotal gastrectomy. She had only abdominal spasm pain and no other abnormalities on imaging or laboratory test results. Hence, the symptoms were very similar to the pain caused by postoperative intestinal adhesion. Imaging examination did not show high-density shadow or presence of liquid or free gas. This likely caused a misdiagnosis of abdominal pain due to intestinal adhesion prior to preoperative consideration.

Most gastrointestinal foreign bodies can be removed by gastroscopy or enteroscopy, only 1% of cases need surgical excision. According to the perforation site and clinical manifestations, treatment is usually chosen through suture perforation site, bowel resection or Hartman procedure.^[9]^ In general, surgeons prefer bowel resection to prevent intestinal fistula caused by inflammation. Because laparoscopic surgery is less traumatic than traditional laparotomy, it has gradually replaced the traditional open-abdominal exploration for the removal of foreign bodies and is now often the surgical approach of choice.^[14]^

In this case, the patient had a misdiagnosis because of the mild presenting symptoms, multiple abdominal surgery history, and negative imaging results. Fortunately, the fish bone did not cause significant damage. Because of the lack of a clear diagnosis before surgery, we preferred laparoscopic surgery. During the operation, we found a small amount of effusion in the pelvic cavity. Given that the intestinal adhesion was present at the upper abdomen, laparoscopy could not provide a comprehensive exploration. Therefore, we opted for open surgery and eventually found the fish bone lodged at the end of the ileum. We chose bowel resection instead of simple suture, because the perforated site was visible with minor fibrinous exudates. The patient recovered well after surgery. The woman recalled that she had eaten fish on the night before the abdominal pain started.

## 4 Conclusion

Accurate diagnosis of complications due to fish bone intake is still difficult, as it is often secondary to the unintentional intake. Therefore, a detailed history of the patient's diet and eating habits is important. Clinical manifestations are mainly determined by the location of perforation which usually occurs at the junction of the ileum and rectal sigmoid colon. Imaging examination and surgery are often used for definite diagnosis.

## Acknowledgments

The authors would like to thank all participants who contributed to this study.

## Author contributions

JCS and WJY reviewed the medical records and scientific literature and wrote the manuscript. YWZ, YW, and CL critically reviewed the manuscript. JSQ and YCF interpreted the CT images. LL, WHW, JDQ, and CWY contributed to the interpretation of data and critically reviewed the manuscript. All authors approved the final version of the manuscript.

**Conceptualization:** Yu Wang.

**Data curation:** Junchuan Song, Weijin Yang, Jiandong Qiu, Jianshen Qiu.

**Formal analysis:** Junchuan Song, Weijin Yang, Nan Lin.

**Funding acquisition:** Yu Wang.

**Investigation:** Nan Lin.

**Software:** Jianshen Qiu.

**Supervision:** Chen Lin.

**Validation:** Weihang Wu, Chen Lin.

**Writing – original draft:** Junchuan Song, Weijin Yang.

**Writing – review & editing:** Junchuan Song, Chen Lin.
